# The relationship between health-related quality of life and social networks among Japanese family caregivers for people with disabilities

**DOI:** 10.1186/1751-0759-2-17

**Published:** 2008-10-01

**Authors:** Hirokazu Arai, Miwa Nagatsuka, Kei Hirai

**Affiliations:** 1 Department of Health Psychology, Osaka University of Human Sciences, Osaka, Japan; 2 National Hospital Organization Osaka Medical Center, Osaka, Japan; 3 Center for the Study of Communication Design, Osaka University, Osaka, Japan; 4 Department of Psychology and Behavioral Sciences Graduate School of Human Sciences, Osaka University, Osaka, Japan; 5 Department of Complementary and Alternative Medicine, Graduate School of Medicine, Osaka University, Osaka, Japan

## Abstract

**Aims:**

The purpose of this study was to examine HRQOL depending on whether the participants have family members with disabilities or not. In addition, we examined the relationship between HRQOL and social networks among family caregivers in Japan.

**Methods:**

The study has a cross-sectional design. Survey forms were distributed to 9205 people aged 30 and older who visited a dispensing pharmacy within fifteen areas of Japan. We collected data on gender, age, job status, and care giving status for persons with disabilities. Moreover, we assessed support size, social support, and HRQOL. Out of the 2029 questionnaires returned, 1763 (male: 663, female: 1100, mean age = 63.06 ± 13.34) were valid for statistical analyses (the available response rate was 19.15%).

**Results:**

A significant difference in HRQOL was identified between family caregivers and non-family caregivers. Further, in males (N = 101), the results confirmed that only social support predicted the PCS and MCS scores, while other variables did not predict either score. On the other hand, in females (N = 144), it was found from the second step of hierarchical multiple regression analysis that only age explained the PCS score, while job status and support size explained the MCS score.

**Conclusion:**

It is reasonable to conclude that the HRQOL of family caregivers was lower than that of non-family caregivers, and that the HRQOL of family caregivers was estimated by their social networks.

## Findings

It is important to assist family members in caring for persons with disabilities. The important role of family caregivers in maintaining their disabled members in the community is becoming increasingly recognized [1]. In addition, Japan has various care requirements for persons with disabilities. It often becomes very important that support is available from family caregivers. Transitional community-based care has increased awareness of the extent of the importance of family caregivers [2].

Caring for persons with disabilities places a chronic physical and mental burden on family caregivers. Thus, it is important that physical, mental and social aspects, in other words, QOL of a family caregiver, are discussed. Canam and Acorn [2] suggest that QOL has emerged as an important concept for determining the impact of community-based care on family caregivers. However, few studies have attempted to explore how the QOL of family caregivers for persons with disabilities is different from the QOL of non-family caregivers. Any potential study should also identify whether there are gender differences in a caregiver's QOL because a caregiver's QOL can be influenced by gender [3].

Some studies have related HRQOL to social networks. Hellström et al. [4] described that the social network determined a high QOL among people aged 75 years and over. Another study has suggested that higher levels of social support increases the self-reported QOL of male workers [5]. Here we show that, as has previously been reported, the QOL of family caregivers might be explained by social network variables.

The purpose of this study was to examine differences in HRQOL depending on whether the participants have family members with disabilities or not. Moreover, we also examined the relationship of HRQOL and social networks among family caregivers.

This study was approved by the institutional review board of the Department of Psychology and Behavioral Sciences, Graduate School of Human Science, Osaka University. The study was a cross-sectional, anonymous mail survey. In this study, we used a convenient sampling technique (e.g. Syad et al., 2008 [6]). The survey forms, "the questionnaire about medicine and lifestyle", were distributed to 9205 people aged 30 and older who visited a dispensing pharmacy within fifteen areas of Japan. These areas included the twelve prefectures in the Kanto, Chubu, Kinki, Chugoku, Shikoku, and Kyushu regions. Staff members in the dispensing pharmacies handed out the questionnaires. If a person who came to a dispensing pharmacy looked like they were over 30 years old, the staff handed the questionnaire to that person. The staff explained the study to the person as follows: 1. Participation in this research is on a voluntary basis. 2. This survey is being conducted on medical care and lifestyle. 3. If you participate in this study by completing a questionnaire, you will receive incentives which include some flower seeds. Moreover, we explained the purpose of the study on the questionnaire and the fact that returning the questionnaire would be regarded as consent for participation, though we asked the participants to return the questionnaires anonymously. The study was carried out from November 2006 to January 2007.

We collected data on the gender, age, and job status of participants. In order to identify family caregivers, we also collected data about whether the participants had family members with disabilities or not. The relevant question was "Are you living with a family member who has a disability?" In this study, we defined somebody as a family caregiver if the response to the question was "Yes".

We used two scales to assess social support that was recognized by participants. One scale was the tangible social support scale [7] to rate support size, i.e. the quantitative amount of social support. The scale was "If you have problems, how many people around you do you have to help you?" The other scale was a social support scale [8], which was altered to suit people of all ages in order to assess the qualitative amount of social support. The scale was "If you have worries or problems how many of your family and friends will listen to you?", and was a 5-point Likert scale. Although these scales have not been validated in a Japanese population, some Japanese studies have used these scales (e.g. Shiozaki et al. [9] and Okabayashi et al. [8])

For this study, we used the Japanese version of the MOS SF-8 which was administered to assess HRQOL. The SF-8 is divided into an 8 dimension health profile (PF, RP, BP, GH, VT, SF, RE, and MH) and 2 summary scores (PCS and MCS). The SF-8 is comprised of 8 items that are assessed by a 5 or 6-point Likert scale. The 8 domain scaled scores range from 0 to 100, with 100 representing optimal health and functioning. The Japanese version of the SF-8 has good reliability and validity among the Japanese population [10].

All data were analyzed using SPSS 15.0J. If missing data were found in the scale, the scores of the corresponding factors were excluded from the analysis. Out of the 2029 questionnaires returned, 1763 were valid for statistical analyses. The available response rate was 19.15% (male: 663, female: 1100, mean age = 63.06 ± 13.34). The rest (n = 266) were invalid due to a lack of major information (gender, age, or care giving status), or because the respondent was below thirty years old.

The results of the chi-squared tests for demographic data showed that more family caregivers were not holding a job than non-family caregivers (care giving status × gender: *χ*^2 ^(1) = 1.47, n.s./care giving status × job status: *χ*^2 ^(1) = 8.00, *p *< .01). The result of a *t*-test identified that the family caregivers' mean age (66.54 ± 12.11) was significantly higher than that of non-family caregivers (62.28 ± 13.48) (*t *(1761) = 5.23, *p *< .001).

With respect to whether the participants were family caregivers or not, the analyses indicated significant differences in all HRQOL scores (Table 1). However, support size and social support were not different in either group.

**Table 1:** Mean (SD) and results of *t*-tests for HRQOL and social network by caregiver status

	family caregivers			non-family caregivers			*t *value	
				
	mean	SD	N	mean	SD	N		
PF	45.31	8.74	291	47.10	7.17	1328	3.70	***
RP	45.95	7.90	295	47.35	7.74	1340	2.80	**
BP	47.14	8.27	308	48.85	8.09	1369	3.33	***
GH	46.96	7.28	302	48.33	6.87	1290	3.07	**
VT	49.09	7.24	306	50.24	6.55	1373	2.71	**
SF	45.09	9.04	302	46.75	8.92	1353	2.92	**
RE	47.33	7.91	300	48.85	6.93	1328	3.34	***
MH	48.49	7.50	307	49.86	6.84	1363	3.11	**
PCS	44.94	7.52	261	46.11	7.20	1172	2.36	*
MCS	48.11	7.59	261	49.29	6.99	1172	2.42	*
support size	3.91	2.75	296	3.96	2.66	1353	0.31	n.s.
social support	3.79	0.74	311	3.80	0.81	1387	0.11	n.s.

*p < .05, **p < .01, ***p < .001

To examine potential factors that explain PCS and MCS scores in men (N = 101) and women (N = 144), two-step hierarchical regression analyses were performed by entering age and job status as a set in the first step, and support size and social support as a set in the second step for males and females (Table 2). In males, the results confirmed that only social support predicted the PCS and MCS scores, while other variables did not predict either score. As for the coefficient of multiple determinations, a significant value was gained with MCS only in the second step. Further, the *R*^2 ^changes identified by the hierarchical regression analysis in the second step were significant in the PCS and MCS scores. On the other hand, in females, it was found from the second step of hierarchical multiple regression analysis that only age explained the PCS score and job status, and support size explained the MCS score. For the coefficient of multiple determinations, a significant value was achieved for PCS and MCS in the first and second steps. The *R*^2 ^change was not significant for either analysis of the female data.

**Table 2:** Result of hierarchical multiple regression to explain PCS and MCS by sex of the family caregiver

PCS: male	β		PCS: female	β	
	step 1	step 2		step 1	step 2
	
age	-.13	-.13	age	-.33***	-.33***
job status	.02	.02	job status	-.03	-.03
support size		.09	support size		-.02
social support		.22*	social support		-.03
	
*R*^2^	.02	.09	*R*^2^	.09***	.08**
*R*^2 ^change		.07*	*R*^2 ^change		.00
	
MCS: male	β		MCS: female	β	
	step 1	step 2		step 1	step 2
	
age	-.07	-.06	age	.18*	.16
job status	-.02	-.02	job status	.20*	.19*
support size		.14	support size		.19*
social support		.25*	social support		.02
	
*R*^2^	-.02	.07*	*R*^2^	.04*	.06*
*R*^2 ^change		.11**	*R*^2 ^change		.04

*p < .05, **p < .01, ***p < .001

One of the important findings that this study identified was a significant difference in HRQOL depending on whether the participants were family caregivers or not. This finding suggests that health care providers should encourage family caregivers to improve their HRQOL more than non-family care givers. Furthermore, there was not a significant difference between family caregivers and non-family caregivers in social network variables.

The second important finding of this study was that the relationship between social networks and HRQOL differed by gender. Specifically, social support explained the PCS and MCS in males, while support size explained the MCS in females. Likewise, according to the present study, *R*^2 ^changes were significant for the MCS in males. From the results of this study, male family caregivers did not necessarily require many supporters to maintain their HRQOL, but rather an attentive listener to their worries or problems. By contrast, the better physical component of female family caregivers was only explained by lower age. Female family caregivers had a preferred mental component if they had a job and many people who support them.

This survey has several limitations. First, because this study was a cross-sectional design, we cannot refer to inferring causal paths. Second, there was a significant difference in HRQOL depending on whether the participants were family caregivers or not, but there were also significant differences in mean age between family caregivers and non-family caregivers. Third, we did not collect data about the degree of care giving for persons with disabilities. Because little research has been directed at evaluating strategies for preserving caregivers physical functioning in addition to their psychological well-being [11], it is very worthwhile to identify social networks as important for the HRQOL of family caregivers. Fourth, in this study, the response rate and R^2 ^values that were significant were relatively low. It should be noted in the interpretation of the results.

In the future, further studies of family caregivers for persons with disabilities should be conducted in detail. For example, research about the specific disability of the family member (e.g. physical disability, mental disabilities, or intellectual disability) should be done. Additionally, we recommend that future research include an investigation of interventions for family caregivers for persons with disabilities to increase support size and social support.

## List of Abbreviations

HRQOL: health-related quality of life; QOL: quality of life; MOS: Medical Outcomes Study; SF-8: Short Form 8-Item Health Survey; PF: physical functioning; RP: role functioning- physical; BP: bodily pain; GH: general health perception; VT: vitality; SF: social functioning; RE: role functioning-emotional; MH: mental health; PCS: summary scores for the physical components of health; MCS: summary scores for the mental components of health.

## Competing interests

The authors declare that they have no competing interests.

## Authors' contributions

HA performed the statistical analysis. All authors contributed to the study design, carried out this study, and approved the final version of this paper.
